# Des masses rénales, pancréatiques et surrénaliennes

**DOI:** 10.11604/pamj.2014.19.184.5393

**Published:** 2014-10-21

**Authors:** Faida Ajili, Mohamed Dridi

**Affiliations:** 1 Service de Médecine Interne, Hôpital Militaire de Tunis, Tunisie; 2 Service d'Urologie, Hôpital Militaire de Tunis, Tunisie

**Keywords:** Von Hippel-Lindau, phacomatose, génétique, Von Hippel-Lindau, phakomatosis, genetic

## Image en medicine

La maladie de Von Hippel-Lindau (VHL) est un syndrome familial rare autosomique dominant associé à des tumeurs malignes ou bénignes surtout des hémangioblastomes rétiniens, cérébelleux, de la moelle épinière, carcinomes à cellules rénales ou phéochromocytomes. Nous rapportons le cas d'une patiente âgée de 35 ans sans antécédents notables, issue d'un mariage non consanguin qui rapporte des douleurs abdominales isolées. L'examen clinique notait une protéinurie à 1+ aux bandelettes urinaires, 2 tâches café au lait centimétriques au niveau de l,abdomen et du cou et une douleur lombaire droite. L,examen neurologique était normal. Il n'y avait pas d'HTA. La biologie notait un SIB. Le scanner abdominal a montré un pancréas siège de multiples masses kystiques intra-glandulaires diffuses dont la plus volumineuse siège en céphalo-isthmique refoulant le cadre duodénal et venant au contact sans envahissement du tronc spléno-mésaraique. Le rein droit est le siège d'une masse tissulaire médio-rénale de 21 mm, se rehaussant de façon hétérogène sans infiltration de la graisse péri rénale. Le rein gauche est le siège de deux masses tissulaires médio-rénale et polaire inférieure de 18*63 mm. Nodule surrénalien gauche spontanément isodense, prenant le contraste et mesurant 17*15 mm. Une phacomatose de VHL était évoquée devant: les masses rénales, les lésions pancréatiques et le nodule surrénalien. L'IRM cérébro-médullaire, le fond d’œil et l'angiographie rétinienne étaient normaux. L’étude génétique a mis en évidence une mutation située sur l'exon 1 du gène VHL. La patiente a eu une tumorectomie droite et une néphrectomie gauche concluant à un carcinome à cellules claires du rein ce qui confortait le diagnostic. Le recul actuel est de 10 mois.

**Figure 1:**
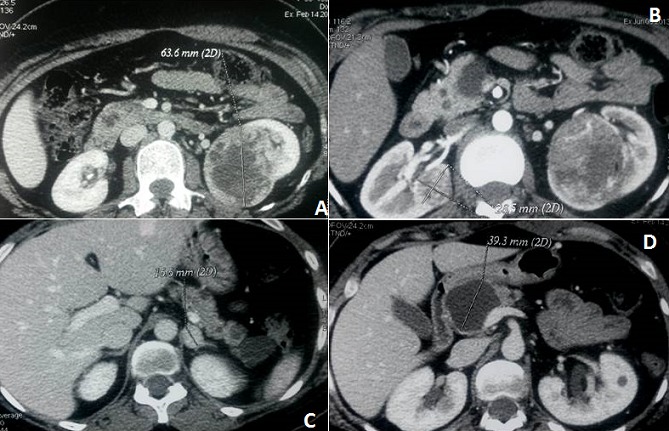
Scanner abdominal des masses rénales (1a-1b), surrénalienne (1c) et pancréatiques (1d)

